# Supplementary material for: Host Protease Activity on Bacterial Pathogens Promotes Complement and Antibiotic-Directed Killing
Source: Pathogens. 2021 Nov 18;10(11):1506. doi: 10.3390/pathogens10111506 (PMC8618517; doi:10.3390/pathogens10111506)
Supplement: Supplementary file 1 [file pathogens-10-01506-s001.zip › pathogens-1458501-supplementary.pdf]

Supplemental Information

Host protease activity on bacterial pathogens promotes complement- and antibiotic-directed killing

Shaorong Chen[1], Dongmei Zhang[1], Alexandria-Jade Roberts[1], Hsueh Chung Lu[1], Carolyn L. Cannon[1], Qing-Ming Qin[1*], Paul de Figueiredo[1,2*]

[1] Department of Microbial Pathogenesis and Immunology, Texas A&M Health Science Center, Bryan, TX 77807

[2] Department of Veterinary Pathobiology, Texas A&M University, College Station, TX 77843

*Address correspondence to Qing-Ming Qin, qmqin@tamu.edu;
Paul de Figueiredo, pjdefigueiredo@tamu.edu

Running title: Protease promotes complement-mediated bacteria-killing

Table S1. Bacterial strains used in this work

Bacterial strain	Features	Reference
Acinetobacter baumannii 5075 (Ab5075)	highly virulent and multi-durg resistant isolate	[1, 2]
Pseudomonas aeruginosa PAO1	virulent	ATCC BAA-47
P. aeruginosa PAO1Δ*Ecotin*	*Ecotin* deleted mutant	Two-Allele Library PW5611
P. aeruginosa PAO1Δ*Wzz*	*Wzz* deleted mutant	Two-Allele Library PW6290
P. aeruginosa PAO1Δ*AprI*	*AprI* deleted mutant	Two-Allele Library PW3254
P. aeruginosa PA-14	Virulent strain	BEI Resource NR-50573
Brucella melitensis 16MΔ*vjbR*	*vjbR* deleted mutant, virulence attenuated	[3]

References

1. Cheng YS, Sun W, Xu M, Shen M, Khraiwesh M, Sciotti RJ, et al. Repurposing Screen Identifies Unconventional Drugs With Activity Against Multidrug Resistant Acinetobacter baumannii. Front Cell Infect Microbiol. 2018;8:438. doi: 10.3389/fcimb.2018.00438.
2. Jacobs AC, Thompson MG, Black CC, Kessler JL, Clark LP, McQueary CN, et al. AB5075, a highly virulent isolate of Acinetobacter baumannii, as a model strain for the evaluation of pathogenesis and antimicrobial treatments. MBio. 2014;5(3).
3. Weeks JN, Galindo CL, Drake KL, Adams GL, Garner HR, Ficht TA. Brucella melitensis VjbR and C12-HSL regulons: contributions of the N-dodecanoyl homoserine lactone signaling molecule and LuxR homologue VjbR to gene expression. BMC Microbiol. 2010;10:167. doi: 10.1186/1471-2180-10-167.

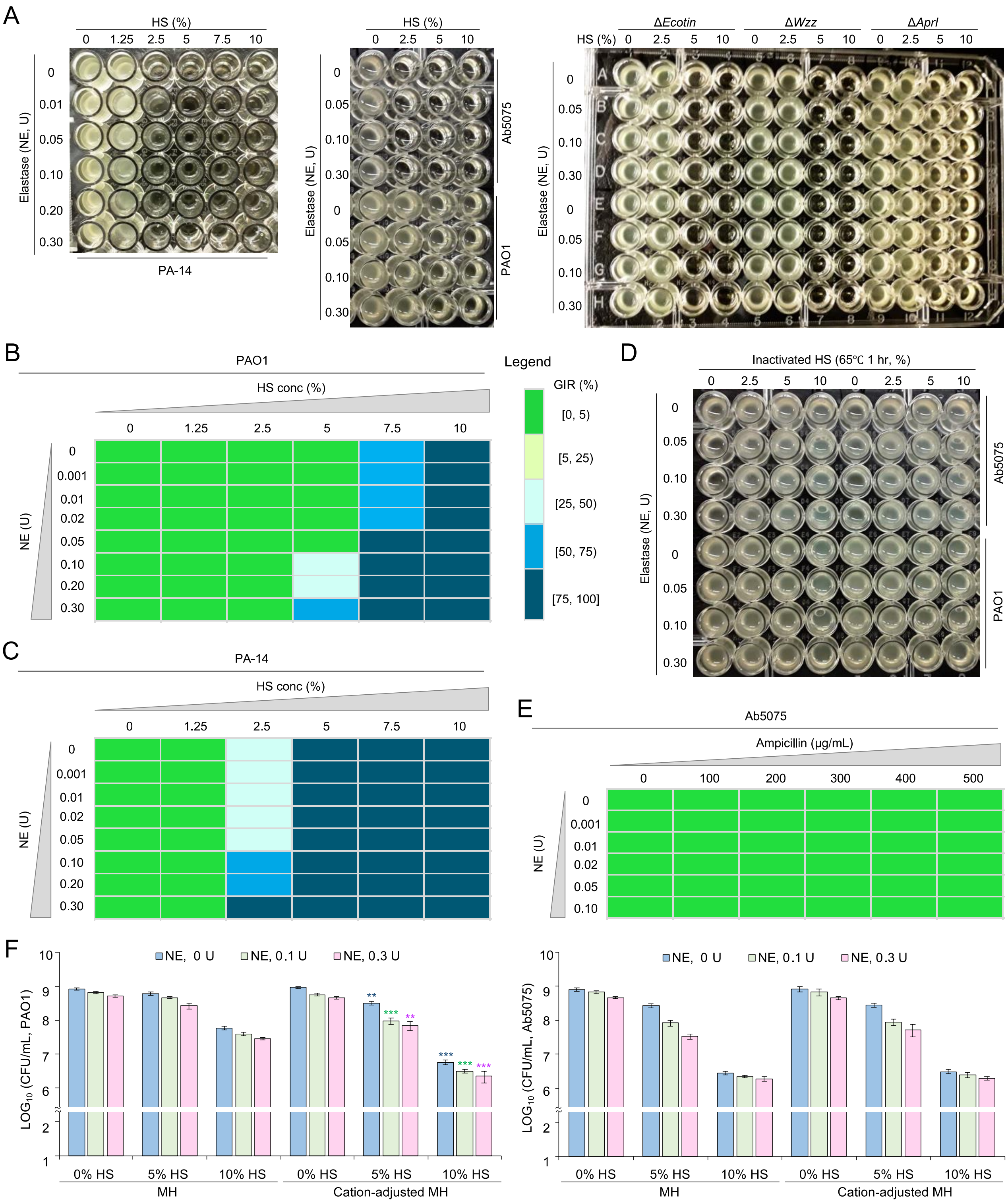

A
HS (%)
0 1.25 2.5 5 7.5 10
Elastase (NE, U)
0 0.01 0.05 0.10 0.20 0.30
PA-14
HS (%)
0 2.5 5 10
Elastase (NE, U)
0 0.05 0.10 0.30 0 0.05 0.10 0.30
Ab5075
PAO1
ΔEcotin ΔWzz ΔAprl
HS (%) 0 2.5 5 10 0 2.5 5 10 0 2.5 5 10
B
PAO1
HS conc (%)
0 1.25 2.5 5 7.5 10
NE (U)
0 0.001 0.01 0.02 0.05 0.10 0.20 0.30
Legend
GIR (%)
[0, 5)
[5, 25)
[25, 50)
[50, 75)
[75, 100]
C
PA-14
HS conc (%)
D
Inactivated HS (65℃ 1 hr, %)
0 2.5 5 10 0 2.5 5 10
E
Ab5075
Ampicillin (μg/mL)
0 100 200 300 400 500
NE (U)
0 0.001 0.01 0.02 0.05 0.10
F
NE, 0 U
NE, 0.1 U
NE, 0.3 U
LOG10 (CFU/mL, PAO1)
LOG10 (CFU/mL, Ab5075)
0% HS 5% HS 10% HS 0% HS 5% HS 10% HS
MH
Cation-adjusted MH

Figure S1. Effect of neutrophil elastase (NE) and human serum (HS) or Ampicillin on bacterial killing. (A) Growth inhibition assays of the bacterial pathogens *Pseudomonas aeruginosa* PA-14, PAO1, and *Acinetobacter baumannii* Ab5075 in the indicated concentrations of NE and HS. (B-C) Combinatorial effect of NE and HS on the tested bacterial strains PAO1 (B) and PA-14 (C). (D) Heat-inactived HS fails to promote NE bacterial killing. (E) NE fails to promote Ab5075 killing in the presence of Ampicillin at the indicated concentrations. Growth inhibition rate (GIR, %) = [(Contrl OD_{600} – treatment OD_{600})/Contrl OD_{600}]×100%. "[" or "]" and "(" or ")" indicate inclusion and exclusion, respectively. conc: concentration. Pictures froms a representative experiments of at least three independent experiments. (F) Bacterial growth inhibition assays for the presence of HS and/or NE in Mueller Hinton broth (MH) or cation-adjusted MH. Data represent mean ± standard error of the mean (SEM) from three independent experiments. **, ***: significance (compared to the same NE and/or HS condition in MH or cation-adjusted MH broth) at $p < 0.01$ and 0.001, respectively.

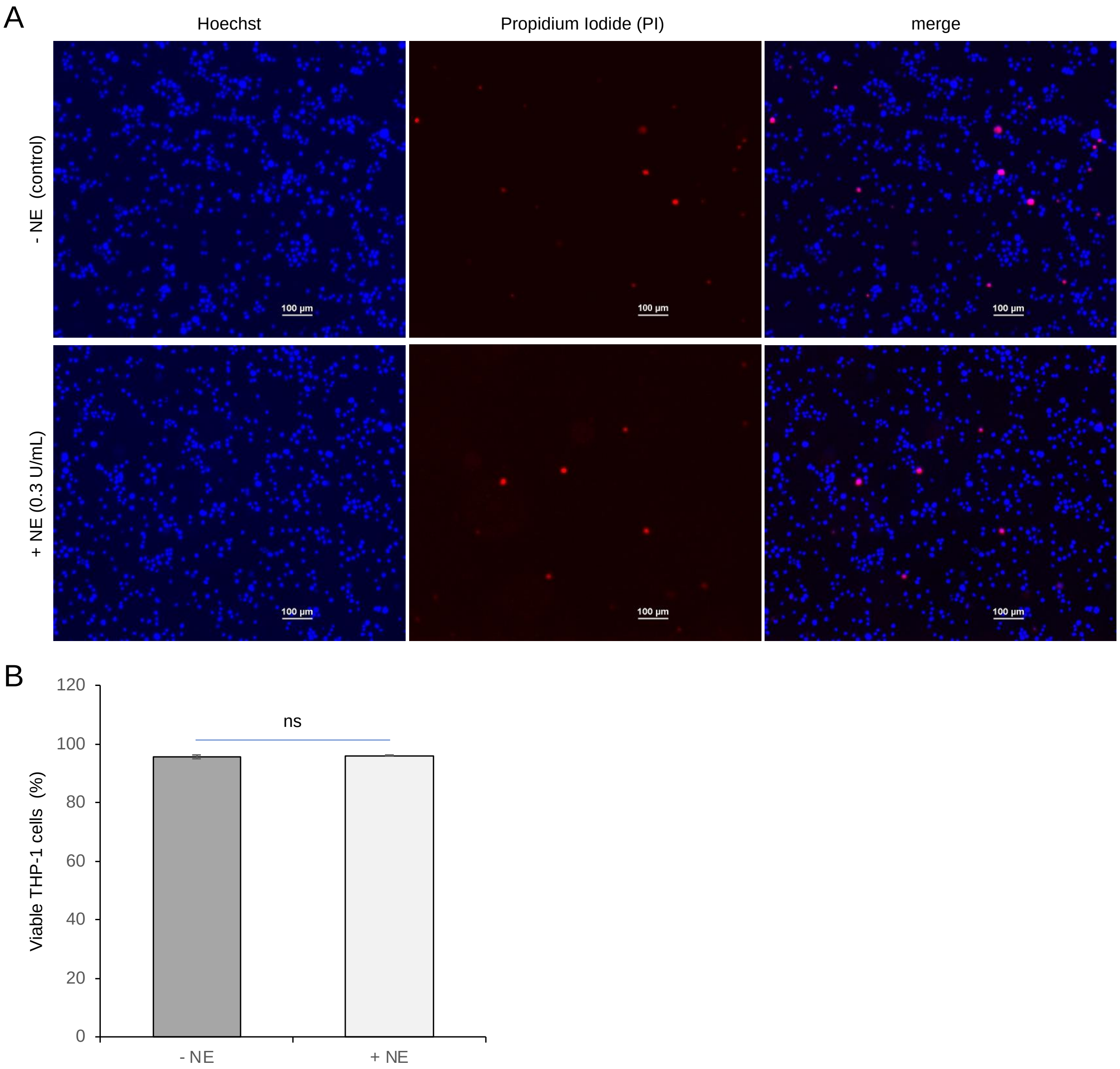

Figure S2. Human neutrophil elastase (NE) does not induce host cell cytotoxicity. Human THP-1 cells were coincubated with (0.3 U/mL) or without NE at at 37℃ for 24 hr. The treated cells were than subjected to staining and fluoresence microscopy assay. (A) Fluoresence images showing viable (blue) and dead (red and purple) THP-1 cells at 24 hr post incubation with or without human NE. (B) Quantification of viable THP-1 cells at 24 hr post incubation with or without NE showing in (A). Images represent one of three independent experiments. Statistical data express as mean ± SEM from three independent experiments. ns: no significan difference.
